# Development and psychometric evaluation of the family intensive care units syndrome inventory

**DOI:** 10.1002/brb3.3101

**Published:** 2023-06-06

**Authors:** Yaser Saeid, Abbas Ebadi, Mohammad Mahdi Salaree, Seyed Tayeb Moradian

**Affiliations:** ^1^ Trauma Research Center and Faculty of Nursing Baqiyatallah University of Medical Sciences Tehran Iran; ^2^ Behavioral Science Research Center Life Style Institute, Faculty of Nursing, Baqiyatallah University of Medical Sciences Tehran Iran; ^3^ Health Research Center, Life style institute and Faculty of Nursing Baqiyatallah University of Medical Sciences Tehran Iran; ^4^ Atherosclerosis Research Center and Faculty of Nursing Baqiyatallah University of Medical Sciences Tehran Iran

**Keywords:** family intensive care units syndrome, family member, intensive care unit, psychometrics

## Abstract

**Background:**

Family members of patient in the intensive care unit (ICU) experience a set of problems which are entitled Family Intensive Care Units Syndrome (FICUS).

**Objectives:**

The aim of this study was to develop and psychometrically evaluate the FICUS Inventory (FICUSI) in Iran.

**Methods:**

This sequential exploratory mixed method study was conducted in 2020 in two main phases. In the first phase, FICUSI was developed based on the results of an integrative review and a qualitative study. In the second phase, the psychometric properties of FICUSI, namely, face, content, and construct validity, reliability, responsiveness, interpretability, and scoring, were evaluated. The sample for the construct validity evaluation consisted of 283 ICU family members.

**Results:**

The primary item pool of FICUSI had 144 items and was reduced to 65 items or omitting overlapping and similar items. The scale‐level content validity index of FICUSI was 0.89. In the construct validity evaluation through exploratory factor analysis, 31 items with factor loading values more than 0.3 were loaded on two factors (namely psychological symptoms and nonpsychological symptoms) which explained 68.45% of the total variance. The Cronbach's alpha and the test‐retest intraclass correlation coefficient of FICUSI were 0.95 and 0.97, respectively.

**Conclusion:**

FICUSI is a valid and reliable instrument which can be used in clinical settings and studies for FICUS assessment. Further studies for the cross‐cultural adaptation of FICUSI in other contexts are recommended.

**Relevance to clinical practice:**

Health care providers in clinical settings can use FICUSI to assess FICUS among the family caregivers of patients in ICU. Health care providers’ better understanding of FICUS helps them understand the quality of their own services for the family members of patients in ICU.

## Graphical abstract

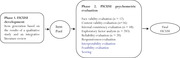

## 1 INTRODUCTION

Many patients are hospitalized each year in the intensive care unit (ICU). The ICU admission rate has increased in recent years due to the increasing prevalence of age‐related problems and chronic conditions (Hawari et al., 2016; Jolivot et al., 2016).

The ICU setting is a complex and stressful environment for the patients and their family members (Beesley et al., 2018). The major sources of stress for the family members of patients in ICU are sudden and unexpected hospitalization of a patient in ICU, unfamiliarity with medical procedures and equipment, implementation of invasive procedures, prolonged ICU stay, and risk of death (McAdam et al., 2010; Schmidt & Azoulay, 2012). The effects of ICU hospitalization on family members are so intense that they may even ignore their own basic needs such as rest, food, and thereby, lose their ability to effectively the problems and strains (Maghsoudi et al., 2007). Ineffective coping with problems can lead to different psychological problems, such as stress, anxiety, despair, depression, and posttraumatic stress disorder for ICU family members (Davidson et al., 2012; Loiselle et al., 2012; Sarhadi et al., 2016). Previous studies reported the high prevalence of health‐related problems, such as anxiety (37%–79%), depression (27%–70.3%), lack of energy, fatigue, grief (52%), and altered quality of life among the family members of patients in ICU (Alfheim et al., 2019; Alfheim et al., 2019; Fumis et al., 2015; Kang et al., 2021; Kentish‐Barnes et al., 2015; McAdam et al., 2010). These problems may start with ICU admission and last for days to months after ICU discharge (Choi et al., 2012; Fumis et al., 2015; McAdam et al., 2010).

In recent years, the term Family Intensive Care Units Syndrome (FICUS) has been used to refer to the problems of the family members of patients in ICU. This syndrome is a set of psychological problems which occur for family members during patient hospitalization in ICU (Davidson et al., 2012; Schmidt & Azoulay, 2012). Recent studies highlighted that FICUS includes not only psychological problems but also physical, spiritual, and social problems (Saeid et al., 2021, 2020). Therefore, FICUS can be a threat to the physical, psychological, spiritual, and social health of the family members of patients in ICU (Saeid et al., 2021).

A key step in FICUS management is its accurate assessment using valid and reliable instruments. Based on the previous definitions of FICUS which mainly addressed psychological problems, previous studies assessed FICUS using psychological assessment instruments, such as the Beck's Depression Inventory, Hospital Anxiety and Depression Scale, and Posttraumatic Stress Disorder Questionnaire (Barth et al., 2016; Bolosi et al., 2018; Davidson et al., 2012). These instruments are not FICUS‐specific and mostly assess psychological problems such as anxiety and depression. The only FICUS‐specific instrument is the Iowa ICU Family Scale which focuses on behavioral responses and does not cover other aspects of FICUS (Halm et al., 1993). Lack of a comprehensive and specific instrument is a major barrier to effective FICUS assessment (Major et al., 2016) and highlights the need for further studies to develop such instruments. The present study tries to narrow this gap. This study was conducted to develop and psychometrically evaluate the FICUS Inventory (FICUSI).

## 2 MATERIALS AND METHODS

### 2.1 Design and setting

**FIGURE 1:**
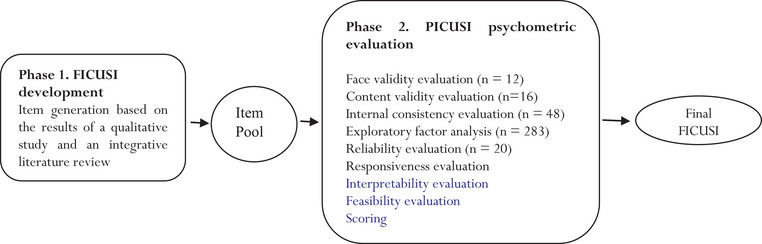
Summary of study method.

This sequential exploratory mixed method study was conducted in 2020 in Iran. The study was conducted in two main phases, namely, of FICUSI development and FICUSI psychometric evaluation (Figure 1).

### 2.2 Phase 1. FICUSI development

The items of the FICUSI were developed through an integrative literature review (Saeid et al., 2020) and a qualitative descriptive study with conventional content analysis (Saeid et al., 2021). In the integrative literature review, FICUS‐related instruments were reviewed and their items were used to generate the FICUSI items. The results of the review and the qualitative study, published elsewhere (Saeid et al., 2022, 2020). First, 144 items were designed based on the findings obtained from the literature review and the qualitative study. Items were carefully assessed respecting their overlaps with each other, meaning, and wording. Overlapping and similar items were combined or omitted and the number of items reduced to 65. Items were scored on a Likert scale as follows: (1) “Never”; (2) “Rarely”; (3) “Sometimes”; (4) “Usually”; and (5) “Always.”

### 2.3 Phase 2. The FICUSI psychometric evaluation

In this phase, the psychometric properties of the FICUSI were assessed. These properties were face, content, and construct validity, reliability, responsiveness, interpretability, and scoring.

#### 2.3.1 Evaluation of face validity

Face validity was evaluated through quantitative and qualitative methods. In the qualitative appraisal of face validity, face‐to‐face cognitive interviews were held with 12 family members of patients in ICU, participants were asked to comment on the difficulty, appropriateness, and clarity of the items and then, necessary revisions were made based on their comments. In the quantitative evaluation of face validity, item impact score was calculated for all items. Accordingly, 12 ICU family members were invited to rate item suitability on a five‐point scale as follows: (5) “Completely suitable”; (4) “Suitable”; (3) “Relatively suitable”; (2) “Slightly suitable”; and (1) “Not suitable at all.” Finally, item impact scores were calculated by multiplying frequency by suitability and items with impact scores higher than 1.5 were considered suitable (Johnson, 2021; Taghizadeh et al., 2017).

#### 2.3.2 Evaluation of content validity

Content validity was also evaluated by quantitative and qualitative methods. In the qualitative content validity assessment, 16 experts in psychology, nursing, and intensive care from Tehran, Iran, were asked to assess and comment on the grammar, wording, allocation, and scores of the items. Necessary revisions were made to the items according to their comments.

In the quantitative content validity evaluation, content validity ratio (CVR) and index (CVI) and modified kappa were calculated. Accordingly, the experts were asked to rate item essentiality on a three‐point scale (“Essential,” “Useful but not essential,” and “Not essential”), and their rating scores were used to calculate CVR. CVI was also determined through asking the experts to rate item relevance on the following four‐point scale: (1) “Irrelevant”; (2) “Somewhat relevant”; (3) “Relevant”; and (4) “Completely relevant.” Then, the CVI of each item was calculated through dividing the number of experts who had rated that item 3 or 4 by the total number of the experts. Items with CVR, CVI, and modified kappa values more than 0.49, 0.78, and 0.74, respectively, were considered acceptable (Ebadi et al., 2017; Polit et al., 2007).

#### 2.3.3 Item analysis

In this step, 48 ICU family members completed the FICUSI and Cronbach's alpha was calculated. Items with inter‐item correlation coefficients more than 0.7 and corrected item‐total correlation coefficients less than 0.3 were considered acceptable (Ebadi et al., 2017).

#### 2.3.4 Evaluation of construct validity

The construct validity of the FICUSI was evaluated by exploratory factor analysis. Accordingly, 283 ICU family members were consecutively recruited from public and private hospitals in Tehran, Iran, to complete the FICUSI and a demographic questionnaire. The demographic questionnaire had items on age, gender, marital status, kinship with patient, financial status, and educational level. A convenience sampling method was applied. Participants were selected from 10 hospitals. Inclusion criteria were Iranian nationality, ability to speak and understand Persian, ability to answer the FICUSI items, and having a patient in ICU and ICU stay more than 48 h. Participants were provided with explanations about the study aim and were asked to complete the study instrument when they were in hospital for patient visitation. Participants who incompletely answered the instrument or voluntarily withdrew from the study were excluded. Sampling adequacy was tested using the Kaiser–Meyer–Olkin test, and a test value of more than 0.7 was interpreted as adequate sample for exploratory factor analysis. The factorability of the items was also tested by the Bartlett's test. Normality was assessed with a skewness of ±3 and a kurtosis of ±7, and the latent factors of the FICUSI were extracted using the maximum likelihood method and promax rotation (Chan & Idris, 2017).

#### 2.3.5 Evaluation of reliability

Reliability was evaluated by evaluating internal consistency, test‐retest stability, and standard error of measurement (SEM). Cronbach's alpha was calculated by internal consistency evaluation and values more than 0.7 were considered acceptable (Taber, 2018). In test‐retest; 12 ICU family members complete the FICUSI twice within a 2‐week interval and then, test‐retest intraclass correlation coefficient (ICC) was calculated and ICC values more than 0.8 were considered acceptable (Liljequist et al., 2019).

#### 2.3.6 Responsiveness

The responsiveness of the FICUSI was evaluated by calculating SEM and minimal detectable changes (MDC) as follows: SEM = SD√1 and MDC = SEM × *z* × 2.

#### 2.3.7 Interpretability

The interpretability of the FICUSI was evaluated using minimally important changes (MIC), ceiling and floor effects, distribution of the total score, and percentage of missing items.

*MIC calculation*: MIC was calculated by multiplying the standard deviation of test‐retest changes by a moderate effect size of 0.5 (Wright et al., 2012). A MIC value greater than MDC confirms interpretability (Ebadi et al., 2017).

*Ceiling and floor effects*: Ceiling and floor effects were calculated for the FICUSI and its factors. Ceiling and floor effects exist when more than 20% of respondents obtain, respectively, the highest and the lowest possible scores of the intended instrument (Ho & Yu, 2015). For calculating the ceiling and floor effects of the FICUSI, the percentage of participants who had obtained, respectively, the highest and the lowest scores was calculated.

*Evaluation of the distribution of the FICUSI score*: The total score of each instrument is expected to vary in different groups such as gender or age groups. In this study, the distribution of FICUSI score was assessed through comparing FICUSI mean score in gender groups.

*Evaluation of the missing items*: The frequency of the missing items was calculated through dividing the number of unresponded items by the total number of the items and multiplying the result by 100 (Dong & Peng, 2013). Missing data were replaced with the mean score.

#### 2.3.8 FICUSI scoring

The FICUSI items were scored on a Likert scale as follows: (1) “Never”; (2) “Rarely”; (3) “Sometimes”; (4) “Usually”; and (5) “Always.” The linear transformation method was used to transform the scores of the FICUSI and its factors to an identical scale in order to facilitate understanding and comparison of the scores. The inventory scores range from 0 to 100. Therefore, higher the FICUSI scores show more severe FICUS. This purpose was made through the following formula:

$$Score=\frac{totalscore-lowestscore}{highestscore-lowestscore}\times 100$$

### 2.4 Ethical considerations

This study was approved by the Ethics Committee of Baqiyatallah University of Medical Sciences, Tehran, Iran (code: IR.BUMS.REC.1396.561). Participants were provided with information about the study aim, voluntariness of participation in and withdrawal from the study, confidentiality of their data, and management of their data only by the authors of the present study. Written informed consent was obtained from all.

### 2.5 Statistical analysis

The SPSS software (v. 24.0) was used to analyze the data. The data were presented through the measures of descriptive statistics (namely frequency, mean, and standard deviation). Data analysis was performed through the Kolmogorov–Smirnov and the independent‐sample *t* tests, exploratory factor analysis, ICC, and Cronbach's alpha.

## 3 RESULTS

### 3.1 Phase 1. FICUSI development

The results of the literature review revealed the lack of a clear definition for FICUS and the limitation of FICUS to psychological symptoms (Saeid et al., 2020). However, the results of our qualitative study showed that the experiences of the family members of patients in ICU can be categorized into four main categories, namely, of threat to psychological well‐being, threat to physical health, threat to social health, and change in spiritual orientation. Therefore, hospitalization in ICU can cause family members not only psychological problems but also physical health problems, alterations in healthy interpersonal relationships, and changes in spiritual beliefs (Saeid et al., 2021).

### 3.2 Phase 2. FICUSI psychometric evaluation

#### 3.2.1 Evaluation of face and content validity

During the evaluation of face validity, the wording of some items was revised to improve their readability and comprehensibility. Moreover, 25 items were omitted due to CVR values less than 0.56, CVI values less than 0.78, or modified kappa values less than 0.74. Accordingly, the numbers of the FICUSI items were reduced to 40. The scale‐level CVI of the FICUSI was 0.89.

#### 3.2.2 Item analysis

All inter‐item correlation coefficients were less than 0.7, whereas five items were omitted due to item‐total correlation coefficients less than 0.3. Therefore, the number of items reduced to 35.

#### 3.2.3 Evaluation of construct validity

A total of 283 family members of patients in ICU completely answered the FICUSI and were included in final analysis. Their mean age was 47.75 ± 15.22 years, and most of them were female (54%) and married (71.4%) (Table 1).

**FIGURE 2:**
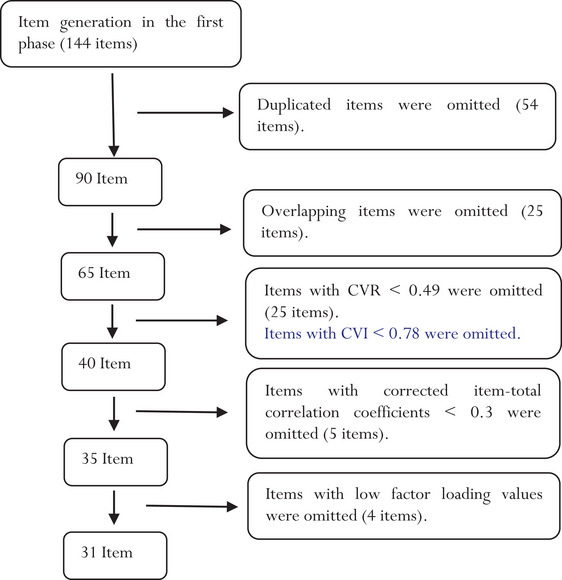
A summary of the instrument and psychometric evaluation.

The value of the Keiser–Meyer–Olkin test was 0.938 and the Bartlett's test was significant (*χ* ^2^ = 5586.88; df = 456; *p* < .001), confirming sampling adequacy and factor analysis appropriateness. Four items were omitted during factor analysis due to factor loading values less than 0.3 and loading under none of the factors. The remaining 31 items were loaded on two main factors with eigenvalues 13.07 and 3.09, respectively. These two factors explained 68.45% of the total variance of FICUS (Table 2 and Figure 2). The two factors were labeled according to the content of their items as psychological symptoms (with 17 items) and nonpsychological symptoms (with 14 items).

#### 3.2.4 Evaluation of reliability

The Cronbach's alpha values of FICUSI and its psychological symptoms and nonpsychological symptoms subscales were 0.95, 0.94, and 0.91, respectively. The test‐retest ICC of FICUSI was also 0.97 (Table 3).

#### 3.2.5 Evaluation of responsiveness

The MDC of FICUSI was 9.33%. An MDC of less than 30% is acceptable, and an MDC of less than 10% is excellent.

#### 3.2.6 Evaluation of interpretability

Based on test‐retest standard deviation, MDC was calculated to be 9.33%. Ceiling and floor effects were 0.7% and 0.4%, respectively, for the FICUSI, 1.8% and 0.4% for its psychological symptom subscale, and 0.7% and 1.4% for its nonpsychological symptom subscale.

Respecting the distribution of the FICUSI score, the mean score of the FICUSI in the 0–100 range was 55.41 among male participants and 53.49 among female participants. Regarding the missing items, findings showed that participants had answered more than 99.9% of the items.

#### 3.2.7 Evaluation of difficulty (feasibility)

The percentage of unresponded items was less than 1%. A robust exploratory factor analysis helped develop a relatively short instrument for FICUS assessment so that the response time of the inventory was 10–15 min. For the difficulty evaluation, participants were asked to document the time spent on completing the instrument. We also documented the time spent on completing the instrument for some participants.

#### 3.2.8 Scoring

The 17‐item psychological symptom subscale of the FICUSI has a total score of 17–85, and the 14‐item nonpsychological symptom subscale has a total score of 14–70. Naturally, after converting the scores to the standard score, the closer an individual's mean score was to 100, the higher scores of the FICUSI show more severe FICUS.

## 4 DISCUSSION

The present study aimed at developing and psychometrically evaluating the FICUSI. Findings showed that the final FICUSI has 31 items in two main subscales, namely, psychological symptoms and nonpsychological symptoms.

The psychological symptoms subscale of FICUSI has seventeen items on the psychological problems associated with patient hospitalization in ICU. The basis of this subscale was the psychological symptoms main category and its emotional distress, hopelessness, changes in sleep pattern, and mood changes subcategories in our qualitative study (Saeid et al., 2021). This subscale addresses psychological symptoms which family members experience following their patients’ hospitalization in ICU. Psychological problems among the ICU family members are more severe and more prevalent than nonpsychological problems. Studies reported anxiety, stress, depression, and sleep disorders as the most prevalent psychological problems among these family members (Choi et al., 2016; Day et al., 2013; McAdam & Puntillo, 2009). Given the higher prevalence of psychological problems among the ICU family members, the number of the items of the psychological symptoms subscale of the FICUSI was greater than the nonpsychological symptoms subscale (17 vs. 14). This highlights the greater importance of the psychological symptoms subscale in explaining FICUS. Of course, the psychological symptoms subscale of the FICUSI is not specific to the assessment of psychological symptoms, such as anxiety, stress, depression, and sleep disorders. Therefore, it cannot be used to comprehensively assess different psychological problems among the ICU family members.

Nonpsychological symptoms were the second subscale of the FICUSI. This subscale addresses the three main categories of the qualitative study, namely, threat to physical health, threat to social health, and change in spiritual orientation (Saeid et al., 2021). The items of this subscale are on the physical, social, and spiritual symptoms associated with patient hospitalization in ICU. Despite the high prevalence of nonpsychological problems of FICUS among the ICU family members, previous studies have paid limited attention, if any, to these problems, though some studies reported that these family members experience physical problems, such as fatigue and sleep disorders (Choi et al., 2014; Choi et al., 2013; Day et al., 2013) as well as social and spiritual problems (Abdel‐Aziz et al., 2017; Gaeeni et al., 2014; Ho et al., 2018; Schleder et al., 2013). However, the FICUSI has different items on both psychological and nonpsychological problems and, hence, is a comprehensive instrument for FICUS assessment.

Psychometric evaluations showed that the FICUSI has acceptable validity and reliability. Our literature searches in both English and Persian databases revealed no FICUS‐specific instrument and hence, the FICUSI is the only valid and reliable instrument in this area. Other instruments which are used for FICUS assessment are neither specific nor comprehensive and focus only on some aspects of FICUS, particularly psychological problems. For example, the Impact of Event Scale (Weiss, 2007) and the Inventory of Complicated Grief (Prigerson et al., 1995) focus on psychological reactions to unexpected events and the Physical Health Questionnaire (Schat et al., 2005) and the Brief Religious Coping measure (Pargament et al., 2011) are not specific to FICUS. All these instruments are general instruments and can be used for different populations of patients and healthy individuals. On the other hand, the Iowa ICU Family Scale, which is the only FICUS‐specific instrument, mostly focuses on behavioral and psychosomatic responses (Halm et al., 1993). Most existing FICUS‐related instruments mainly address psychological symptoms and, hence, are not comprehensive. However, the FICUSI is a FICUS‐specific instrument and addresses the different aspects of FICUS. Comprehensive assessment of FICUS using the FICUSI can provide the necessary data for effective FICUS management.

The scale‐level CVI of the FICUSI was 0.89, confirming the acceptable content validity of the instrument. Moreover, the two factors of the FICUSI explained 68.45% of the total variance of FICUS which is acceptably high (Ho, 2013). The Cronbach's alpha values of the FICUSI and its two subscales as well as the test‐retest ICC of the instrument were also greater than 0.90, confirming its high reliability. Other psychometric properties of the FICUSI (including responsiveness and interpretability) were also acceptable.

### 4.1 Limitations

One of the limitations of the present study was our limited access to eligible ICU family members which prolonged the process of sampling. Moreover, the FICUSI was developed and psychometrically evaluated in the sociocultural context of Iran and hence, cross‐cultural adaptation of the inventory in other contexts is necessary.

The other of limitation was failure to performed confirmatory factor analysis because coronaviruses disease (COVID‐19) pandemic and the impossibility of access to family members.

### 4.2 Implications for critical care nursing practice and research

Critical care nurses in clinical settings can use the FICUSI to assess FICUS among the ICU family members. Increasing critical care nurses’ awareness and understanding of FICUS helps them understand the quality of their own services for the ICU family members.

Based on the results of the FICUSI, health care providers can develop appropriate strategies to improve the experiences of these family members. Moreover, researchers can use the FICUSI to assess FICUS and monitor the effects of the available guidelines and their interventions on FICUS. Furthermore, the FICUSI can be used to assess the educational needs of nurses and other health care staff in ICU to develop and provide FICUS‐related training.

## 5 CONCLUSION

The FICUSI is a valid and reliable FICUS‐specific instrument for comprehensive assessment of psychological and nonpsychological aspects in Iran. This instrument can also be used to assess FICUS changes over time.

### 5.1 What is known about the Topic?

■ FICUS is a major threat to health among the family members of patients in ICU.
■ A key step in FICUS management is its accurate assessment using valid and reliable instruments.
■ Lack of a comprehensive and specific instrument for FICUS assessment is a major barrier to effective FICUS assessment and management.

### 5.2 What this paper adds?

■ Psychometric evaluations showed that the FICUSI has acceptable validity and reliability.
■ The nurses and researchers can use the FICUSI to assess FICUS and monitor the effects of the available guidelines and their interventions on FICUS.
■ Based on the results of the FICUSI, health care providers can develop appropriate strategies to improve the experiences of these family members.
■ The results of the FICUSI application can be used to develop more effective programs and interventions for FICUS management.

## AUTHOR CONTRIBUTIONS

All authors contributed to making this manuscript: Yaser Saeid and Seyed Tayeb Moradian contributed to analysis, writing, data collection, and literature research; Mohammad Mahdi Salaree and Abbas Ebadi contributed to data collection, literature research, and some writing. All authors contributed to the discussion and have seen and approved the final version of the study.

## CONFLICT OF INTEREST STATEMENT

We declare that we have no conflict of interests.

### PEER REVIEW

The peer review history for this article is available at https://publons.com/publon/10.1002/brb3.3101.

6

**TABLE 1:** Participants’ demographic questionnaire

Characteristics		*N* (%)
Gender	Male	127 (46)
	Female	149 (54)
Marital status	Single	71 (25.7)
	Married	197 (71.4)
	Divorced	8 (2.9)
Educational level	Guidance school	91 (33)
	Diploma	68 (24.6)
	Associate diploma	31 (11.2)
	Bachelor's	60 (21.7)
	Master's	26 (9.4)
Financial status	Poor	53 (19.2)
	Medium	124 (70.3)
	Good	98 (10.1)
Kinship with patient	Parent	27 (9.7)
	Child	103 (37.3)
	Sibling	54 (19.6)
	Other	55 (19.9)

**TABLE 2:** The results of exploratory factor analysis to determine the factor structure of Family Intensive Care Units Syndrome Inventory (FICUSI)

No.	Items	Factor loading		Cronbach's alpha	Mean (SD)
		1	2		
1	I have stress	0.748	—	0.92	3.95 (0.99)
2	I feel worried	0.845	—	0.919	3.85 (0.67)
3	I have anxiety	0.907	—	0.921	3.20 (1.06)
4	I have become sensitive and irritable	0.809	—	0.917	3.25 (0.91)
5	I feel restless	0.844	—	0.919	3.35 (1.13)
6	I have become impatient	0.809	—	0.920	3.10 (1.11)
7	I do not have concentration	0.736	—	0.916	3.20 (0.89)
8	I have become forgetful	0.416	—	0.918	2.85 (1.22)
9	My thoughts have become disturbed	0.670	—	0.917	3 (1.17)
10	I am shocked	0.624	—	0.915	3.05 (1.14)
11	I feel baffled	0.667	—	0.916	3.25 (0.85)
12	I have fear over the occurrence of adverse events	0.753	—	0.917	3.70 (1.03)
13	I have fear over losing my patient	0.541	—	0.921	3.85 (1.13)
14	I easily lose my temper	0.537	—	0.915	3.15 (0.98)
15	I feel alone	0.448	—	0.917	3.05 (0.88)
16	My sorrow and unhappiness have increased	0.657	—	0.920	3.50 (1.14)
17	I do not have a comfortable sleep	0494	—	0.916	3.05 (1.14)
18	I do not anymore enjoy the things which were previously enjoyable	—	0.488	0.919	3.35 (0.74)
19	I have pangs of conscience	—	0.566	0.919	2.15 (1.09)
20	I quickly get tired	—	0.442	0.928	3.25 (1.02)
21	I have developed physical problems	—	0.758	0.919	3.20 (0.95)
22	I do not have enough energy	—	0.608	0.922	2.60 (0.88)
23	My appetite has changed	—	0.465	0.918	2.65 (1.13)
24	My attention to personal hygiene (bathing, brushing, etc.) has reduced	—	0.749	0.920	3.20 (0.83)
25	I have developed problems in establishing interpersonal communication	—	0.656	0.917	3.25 (0.91)
26	My occupational and living plans have been messed up	—	0.457	0.916	3.05 (1.09)
27	My attention to other family members has reduced	—	0.641	0.928	2.45 (1.05)
28	I feel empty	—	0.849	0.918	2.55 (0.82)
29	Life has become meaningless for me	—	0.711	0.920	2.80 (1.15)
30	I think God has forgotten me	—	0.773	0.918	3.45 (0.99)
31	My reliance on God and trust in Him have reduced	—	0.636	0.923	3.50 (0.94)
	**Variance %**	36.65	31.80		
	**Cumulative variance %**	68.45			

**TABLE 3:** The results of Family Intensive Care Units Syndrome Inventory (FICUSI) reliability assessment

Factors	Mean ± SD	ICC	95% CI	SEM	MDC	MDC %	Agreement
Psychological threat	56.80 ± 12.11	0.98	0.96–0.99	1.70	4.71	8.29	Excellent
Nonpsychological threat	39.95 ± 8.67	96/0	0.90–0.98	‍73/1	79/4	98/11	Satisfactory
Total	96.75 ± 18.88	97/0	0.95–0.99	26/3	03/9	33/9	Excellent

Abbreviations: MDC, minimal detectable changes; SEM, standard error of measurement.
